# Glycohemoglobin: A new warning strategy for non-alcoholic fatty liver disease: Study from the NHANES 2017- 2020

**DOI:** 10.3389/fendo.2022.1078652

**Published:** 2022-12-22

**Authors:** Jiaxing Hou, Yanyu Liu, Zhen Deng, Jichun Sun, Mingyi Zhao

**Affiliations:** ^1^ Department of Hepatopancreatobiliary Surgery, The Third Xiangya Hospital of Central South University, Changsha, China; ^2^ Department of Science and Education, Changsha Central Hospital Affiliated to University of South China, Changsha, China; ^3^ Department of Pediatrics, The Third Xiangya Hospital of Central South University, Changsha, China

**Keywords:** glycohemoglobin, median LSM, liver stiffness, NAFLD, NHANES

## Abstract

**Context:**

The development and progression of Non-alcoholic Fatty Liver Disease (NAFLD) is associated with type 2 diabetes mellitus (T2DM), but there are no studies to demonstrate whether blood glucose levels are associated with the progression of NAFLD.

**Objective:**

Alterations in glucose metabolism may cause hepatic steatosis and inflammatory responses, leading to hepatocyte damage and promoting NAFLD’s progression. Since glycohemoglobin reflects current blood glucose levels and is easily detectable, the present study aimed to investigate whether glycohemoglobin is associated with liver stiffness in patients with NAFLD.

**Methods:**

We studied 1510 NAFLD patients aged 20-80 in NHANES 2017- March 2020, who were defined using the controlled attenuation parameter (CAP) ≥263 dB/m. Multivariable linear regressions were used to assess the independent association between glycohemoglobin and median liver stiffness measurements (LSM) after adjusting for potential confounders. Subsequently, they were subjected to curve fitting and threshold effect analysis. Stratified analysis was used to find the variables affecting the relationship.

**Results:**

Glycohemoglobin and median LSM showed a positive correlation in different models (β (95% CI): Crude Model: 1.460 (1.053, 1.867); Model 1: 1.476 (1.066, 1.885); Model 2: 1.517 (0.919, 2.115)), and this correlation disappeared when glycohemoglobin ≥8.6%. Furthermore, this correlation was more pronounced in the non-diabetic and former smoking subgroups.

**Conclusion:**

In patients with NAFLD, glycohemoglobin may reflect the degree of liver stiffness, and preventing excessive glycohemoglobin may have a positive effect on slowing the progression of NAFLD especially in non-diabetic and former smokers.

## 1 Introduction

Non-alcoholic fatty liver disease (NAFLD) is the liver manifestation of metabolic syndrome and is the most common cause of liver disease worldwide (1). The global prevalence of NAFLD is estimated to be about 25%, affecting more than 80 million people in the United States alone (2). It is subdivided into two primary subtypes, non-alcoholic fatty liver (NAFL) and non-alcoholic steatohepatitis (NASH). The NASH evolved from simple hepatic steatosis to inflammation and ballooning and then progressed to fibrosis, eventually leading to cirrhosis and hepatocellular carcinoma (HCC) (3). In the US, the proportion of NASH as a potential cause of HCC increased 7.7-fold (from 2.1% to 16.2%) in patients listed for liver transplantation (4). Liver biopsy is the gold standard for identifying NAFL and NASH, but it is unsuitable for large-scale use due to its high price, subjectivity, and invasiveness (5, 6). The ultrasound-based elastography techniques use ultrasound to detect the velocity of the shear waves in liver tissue and convert it into liver stiffness measurements (LSM) (7). LSM is gradually becoming a common method for assessing liver stiffness due to its non-invasive (8, 9).

Many studies have shown that NAFLD is closely associated with insulin resistance and type 2 diabetes mellitus (T2DM) (10–13). Patients with NAFLD are more likely to develop diabetes than those without NAFLD (14). When insulin resistance occurs, it causes changes in glucose metabolism, which leads to hepatic steatosis. The combined effects of hepatic steatosis, lipotoxicity and inflammation cause steatohepatitis and hepatocyte injury (ballooning). When mitochondrial dysfunction occurs, it can cause oxidative stress, and they can promote each other. The combination of these factors causes hepatocyte death (Necrosis, Necroptosis and Apoptosis), which activates hepatic stellate cells and causes collagen deposition. Ultimately, this leads to fibrosis, cirrhosis and even HCC (15). Glycohemoglobin can reflect recent blood glucose levels and glucose metabolism (16). Therefore, we speculate that glycohemoglobin may serve as a potential marker of NAFLD progression. However, there is no in-depth study on the association between glycohemoglobin and LSM in NAFLD.

Therefore, the present study used multiple linear regression to obtain evidence of a possible association between glycohemoglobin and LSM in men and women aged 20-80 from the National Health and Nutrition Examination Survey (NHANES) and compared the differences between those with and without diabetes. Curve fitting and threshold effects analysis were used to further validate their relationship. Stratified analysis was used to find potential factors that affect this relationship.

## 2 Method

### 2.1 Data source and study population

**Figure 1:**
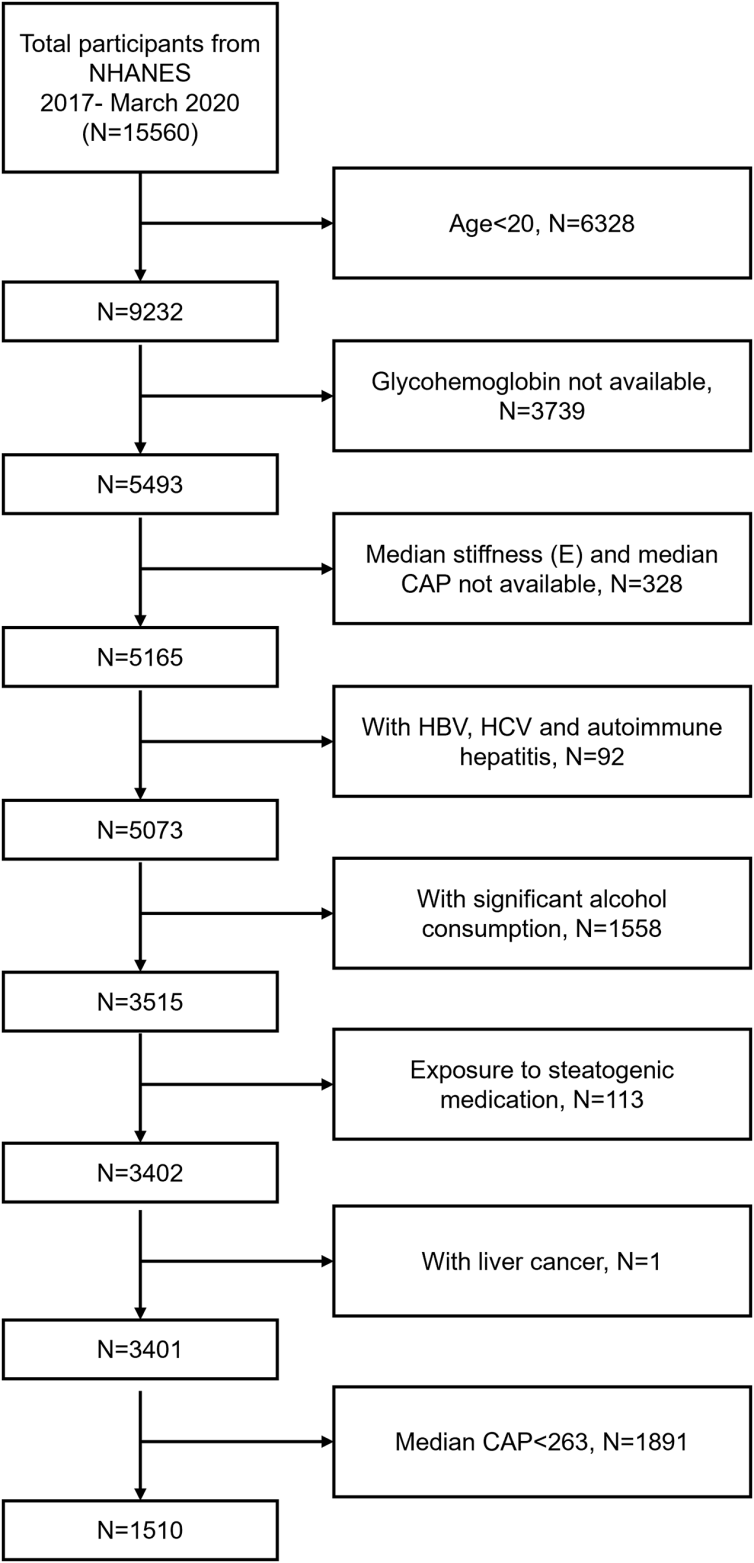
Flow chart of participants selection. NHANES, National Health and Nutrition Examination Survey; CAP, Controlled Attenuation Parameter; HBV, Hepatitis B Virus; HCV, Hepatitis C Virus.

NHANES is a cross-sectional study conducted by the National Center for Health Statistics of the Centers for Disease Control and Prevention in the United States. The goal of NHANES is to provide objective statistics on health problems and to address public health problems that arise among the public. The data analyzed in this study was from 2017-March 2020 cycle of the NHANES. Among the 15560 participants, we excluded 6328 participants with age <20 years, 3739 participants with missing glycohemoglobin data, 328 participants with missing median stiffness (E) and median controlled attenuation parameter (CAP) data, 92 participants with HBV (by self-reported questionnaire), HCV (by the presence of hepatitis C antibodies) or autoimmune hepatitis (by self-reported questionnaire), 1558 participants with significant alcohol consumption (>30g/d in men and >20 g/d in women), 113 participants exposed to steatogenic medication (amiodarone, valproate, methotrexate, tamoxifen, and corticosteroid) for more than 6 months, 1 participant with liver cancer, and 1891 participants with median CAP<263 dB/m. Finally, 1510 participants were enrolled in the study (**Figure 1**).

### 2.2 Study variables and definition of NAFLD

Marital status was divided into married or cohabiting with a partner vs. others. According to the ratio of family income-to-poverty, the economic status was divided into 0.99 (below poverty) or 1.00 (at or above poverty). Educational status was divided into high school graduation and non-high school graduation. Alcohol consumption was assessed using a self-reported questionnaire based on the amount and frequency of drinking. Never smokers were defined as participants who answered "Do you smoke at least 100 cigarettes in life?" with "No", those who answered "Yes" were then asked "Do you now smoke cigarettes?" and when they answered "Not at all" they were classified as former smokers, and those who answered "Every day" or "Some days" were categorized as current smokers. Hypertension was classified as systolic blood pressure ≥140 mmHg or diastolic pressure ≥90 mm Hg and/or current with antihypertensive drug treatment. Diabetes was defined as fasting plasma glucose levels ≥126 mg/dL, glycohemoglobin ≥6.5%, or with hypoglycemic drugs or insulin treatment (17). Physical activity representing moderate or vigorous intensity leisure-time, occupation-related, and transportation-related physical activity in a typical week. The first day’s total alcohol consumption data was used to estimate the alcohol use. The detection and calculation methods of age, gender, ethnicity, body mass index, alanine aminotransferase, aspartate aminotransferase, gamma glutamyl transferase, alkaline phosphatase, total cholesterol, total bilirubin, blood urea nitrogen, uric acid and glycohemoglobin are available on the NHANES website (https://www.cdc.gov/nchs/nhanes/). CAP and LSM were obtained by FibroScan^®^ model 502 V2 Touch. NAFLD was defined as CAP score of 263 dB/m or more (S1, the cutoff of sensitivity fixed at 90%) (18).

### 2.3 Statistical analysis

All analyses were conducted using R (http://www.r-project.org) and EmpowerStats (http://www.empowerstats.com). Appropriate weighting for each analysis was used due to the complex survey design of the NHANES. We described Mean ± SD of the continuous variables and frequencies of the categorical variables. Multivariable linear regressions were performed to determine an independent association between glycohemoglobin and median LSM after adjusting for potential confounders. Two-piecewise linear regression model was used to examine the saturation effect. Then subgroup analyses were performed. Smooth curve fitting was conducted to deal with non-linearity.

## 3 Results

The baseline characteristics of participants according to diabetes are shown in **Table 1**. Compared with the non-diabetic participants (N=1113), the diabetic participants (N=397) had higher glycohemoglobin and median LSM (7.535 ± 1.777 vs. 5.526 ± 0.386) (7.757 ± 5.986 vs. 6.182 ± 5.076). The daily alcohol consumption of the non-diabetic participants was higher than that of the diabetic participants (10.618 ± 29.828 vs. 4.436 ± 19.762). In terms of total cholesterol and blood urea nitrogen, the non-diabetic participants were higher than the diabetic participants. In addition, they also showed a higher rate of marriage, physical activity, and never smoking. The prevalence of hypertension in diabetic participants was higher than that in non-diabetic participants (45.185% vs. 39.304%).

**Table 1:** Weighted characteristic of the participants according to diabetes.

	Non-diabetic (N=1113)	Diabetic (N=397)	*P* value
Glycohemoglobin, %	5.526 ± 0.386	7.535 ± 1.777	<0.00001
Median LSM, kpa	6.182 ± 5.076	7.757 ± 5.986	<0.00001
Age, y	51.384 ± 18.452	50.486 ± 18.261	0.43203
Body mass index, kg/m^2^	30.285 ± 7.545	30.723 ± 7.624	0.35148
Alcohol intake, g/d	10.618 ± 29.828	4.436 ± 19.762	0.00032
ALT, u/l	21.069 ± 13.798	20.527 ± 15.246	0.53832
AST, u/l	21.179 ± 10.774	20.800 ± 11.004	0.57284
GGT, iu/l	26.608 ± 25.911	29.872 ± 35.430	0.06625
ALP, iu/l	77.371 ± 23.949	79.338 ± 25.291	0.19209
Total bilirubin, umol/l	7.863 ± 5.075	7.297 ± 3.996	0.05884
Total cholesterol, mmol/l	4.796 ± 0.994	4.645 ± 1.146	0.01946
Blood Urea Nitrogen, mmol/l	5.490 ± 2.002	5.147 ± 2.002	0.00583
Uric Acid, umol/l	325.593 ± 92.316	319.423 ± 77.648	0.26309
Ethnicity, %			0.54820
Mexican American	8.164	10.267	
Other Hispanic	9.139	10.443	
Non-Hispanic White	57.902	53.916	
Non-Hispanic Black	14.778	15.862	
Other Race	10.017	9.511	
Male	46.804	43.106	0.20995
Married status, %	56.329	49.550	0.02144
Poverty, %	26.115	24.958	0.65514
High education, %	84.179	80.290	0.07887
Physical activity, %	72.803	59.798	<0.00001
Hypertension, %	39.304	45.185	0.04324
Smoking, %			<0.00001
Current smoker	21.022	12.489	
Former smoker	23.303	35.801	
Never smoker	55.675	51.710	

Mean ± SD for continuous variables; % for categorical variables. LSM, Liver Stiffness Measurements; ALT, Alanine Aminotransferase; AST, Aspartate Aminotransferase; GGT, Gamma Glutamyl Transferase; ALP, Alkaline Phosphatase.

We performed a multivariable linear regression analysis of glycohemoglobin and median LSM (**Table 2**). Glycohemoglobin and median LSM had a positive correlation in Crude Model (β=0.499, P<0.0001) and Model 1 (β=0.500, P<0.00001), adjusted age, gender, and ethnicity. However, the positive correlation between glycohemoglobin and median LSM did not reach statistical significance in Model 2 (β=0.275, P=0.07124), adjusted age, gender, ethnicity, body mass index, marital status, poverty, high education, physical activity, hypertension, smoking, alcohol use, alanine aminotransferase, aspartate aminotransferase, gamma glutamyl transferase, alkaline phosphatase, total cholesterol, total bilirubin, blood urea nitrogen and uric acid. After transforming glycohemoglobin into categorical variables, we found that median LSM tended to increase when glycohemoglobin increased in these three models (P-trend<0.001).

**Table 2:** The correlation between glycohemoglobin and median LSM.

	Crude Model β (95% CI) *P* Value	Model 1 β (95% CI)*P* Value	Model 2β (95% CI)*P* Value
Glycohemoglobin, %	0.499 (0.281, 0.717) <0.00001	0.500 (0.282, 0.718) <0.00001	0.275 (-0.024, 0.574) 0.07124
Glycohemoglobin, % (Quartile)			
Q1	0	0	0
Q2	0.091 (-0.711, 0.892) 0.82478	0.070 (-0.735, 0.875) 0.86493	0.162 (-0.654, 0.978) 0.69708
Q3	0.612 (-0.180, 1.403) 0.12997	0.620 (-0.176, 1.415) 0.12710	0.617 (-0.192, 1.426) 0.13519
Q4	2.470 (1.681, 3.258) <0.00001	2.481 (1.690, 3.271) <0.00001	2.860 (1.694, 4.027) <0.00001
*P*-trend	<0.001	<0.001	<0.001

Crude Model: No covariates were adjusted. Model 1: Age, gender and ethnicity were adjusted. Model 2: Age, gender, ethnicity, body mass index, marital status, poverty, high education, physical activity, hypertension, smoking, alcohol use, alanine aminotransferase, aspartate aminotransferase, gamma glutamyl transferase, alkaline phosphatase, total cholesterol, total bilirubin, blood urea nitrogen and uric acid were adjusted. LSM, Liver Stiffness Measurements; CI, Confidence Interval.

**Figure 2:**
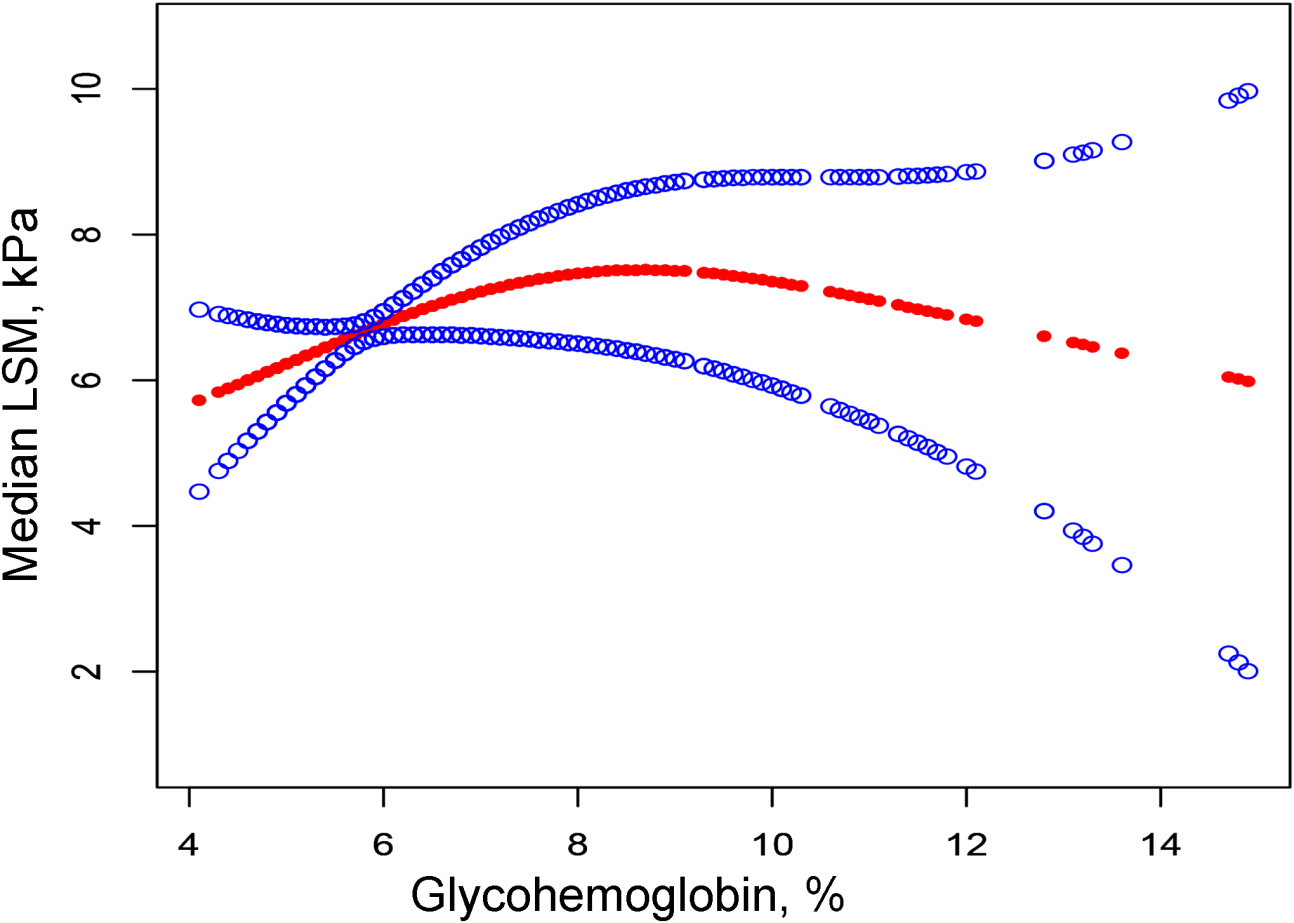
The correlation between glycohemoglobin and median LSM. The red line represents the smooth curve. The blue lines represent the 95% confidence interval. Age, gender, ethnicity, body mass index, marital status, poverty, high education, physical activity, hypertension, smoking, alcohol use, alanine aminotransferase, aspartate aminotransferase, gamma glutamyl transferase, alkaline phosphatase, total cholesterol, total bilirubin, blood urea nitrogen and uric acid were adjusted. LSM, Liver Stiffness Measurements.

Then we performed the adjusted smooth curve of the glycohemoglobin and median LSM (**Figure 2**). In the first half of the curve, the median LSM increases with the increase of glycohemoglobin, and this trend gradually disappears in the second half of the curve. Then through the two-piecewise linear regression model, we found a turning point at 8.6%. **Table 3** shows the saturation effect analysis of glycohemoglobin on median LSM. It can be seen that when glycohemoglobin<8.6%, glycohemoglobin was positively correlated with median LSM in the three models (Crude Model: β=1.460, P<0.00001; Model 1: β=1.476, P<0.00001; Model 2: β=1.517, P<0.00001), when glycohemoglobin ≥ 8.6%, the positive correlation disappeared (Crude Model: β=-0.783, P=0.04261; Model 1: β=-0.631, P=0.15175; Model 2: β=-0.843, P=0.12593). And then, glycohemoglobin was converted into a classification variable for sensitivity analysis. In the three models, when glycohemoglobin<8.6%, with the increase of glycohemoglobin, the median LSM gradually increased (P-trend<0.001). However, when the glycohemoglobin ≥ 8.6%, the median LSM shows a downward trend with the increase of glycohemoglobin (Crude Model: P -trend=0.012; Model 1: P -trend=0.029; Model 2: P -trend=0.072).

**Table 3:** Saturation effect analysis of glycohemoglobin on median LSM.

	Crude Model	Model 1	Model 2
	β (95% CI) *P* Value	β (95% CI) *P* Value	β (95% CI ) *P* Value
Glycohemoglobin			
<8.6%	1.460 (1.053, 1.867) <0.00001	1.476 (1.066, 1.885) <0.00001	1.517 (0.919, 2.115) <0.00001
>=8.6%	-0.783 (-1.527, -0.039) 0.04261	-0.631 (-1.485, 0.222) 0.15175	-0.843 (-1.905, 0.219) 0.12593
Glycohemoglobin<8.6% (Quartile)			
Q1	0	0	0
Q2	0.071 (-0.826, 0.968) 0.87700	0.054 (-0.846, 0.955) 0.90572	0.028 (-0.876, 0.933) 0.95151
Q3	0.450 (-0.316, 1.216) 0.24955	0.442 (-0.329, 1.212) 0.26143	0.413 (-0.375, 1.200) 0.30450
Q4	2.421 (1.612, 3.230) <0.00001	2.450 (1.637, 3.263) <0.00001	2.397 (1.302, 3.491) 0.00002
*P*-trend	<0.001	<0.001	<0.001
Glycohemoglobin>=8.6% (Quartile)			
Q1	0	0	0
Q2	-1.311 (-4.834, 2.212) 0.46803	-1.181 (-4.859, 2.498) 0.53154	-2.234 (-6.796, 2.328) 0.34191
Q3	-3.275 (-6.505, -0.046) 0.05066	-3.395 (-6.726, -0.063) 0.04994	-3.424 (-7.249, 0.401) 0.08560
Q4	-3.962 (-7.428, -0.496) 0.02817	-3.453 (-7.068, 0.162) 0.06562	-3.634 (-8.453, 1.185) 0.14578
*P*-trend	0.012	0.029	0.072

Crude Model: No covariates were adjusted. Model 1: Age, gender and ethnicity were adjusted. Model 2: Age, gender, ethnicity, body mass index, marital status, poverty, high education, physical activity, hypertension, smoking, alcohol use, alanine aminotransferase, aspartate aminotransferase, gamma glutamyl transferase, alkaline phosphatase, total cholesterol, total bilirubin, blood urea nitrogen and uric acid were adjusted. LSM, Liver Stiffness Measurements; CI, Confidence Interval.

**Figure 3:**
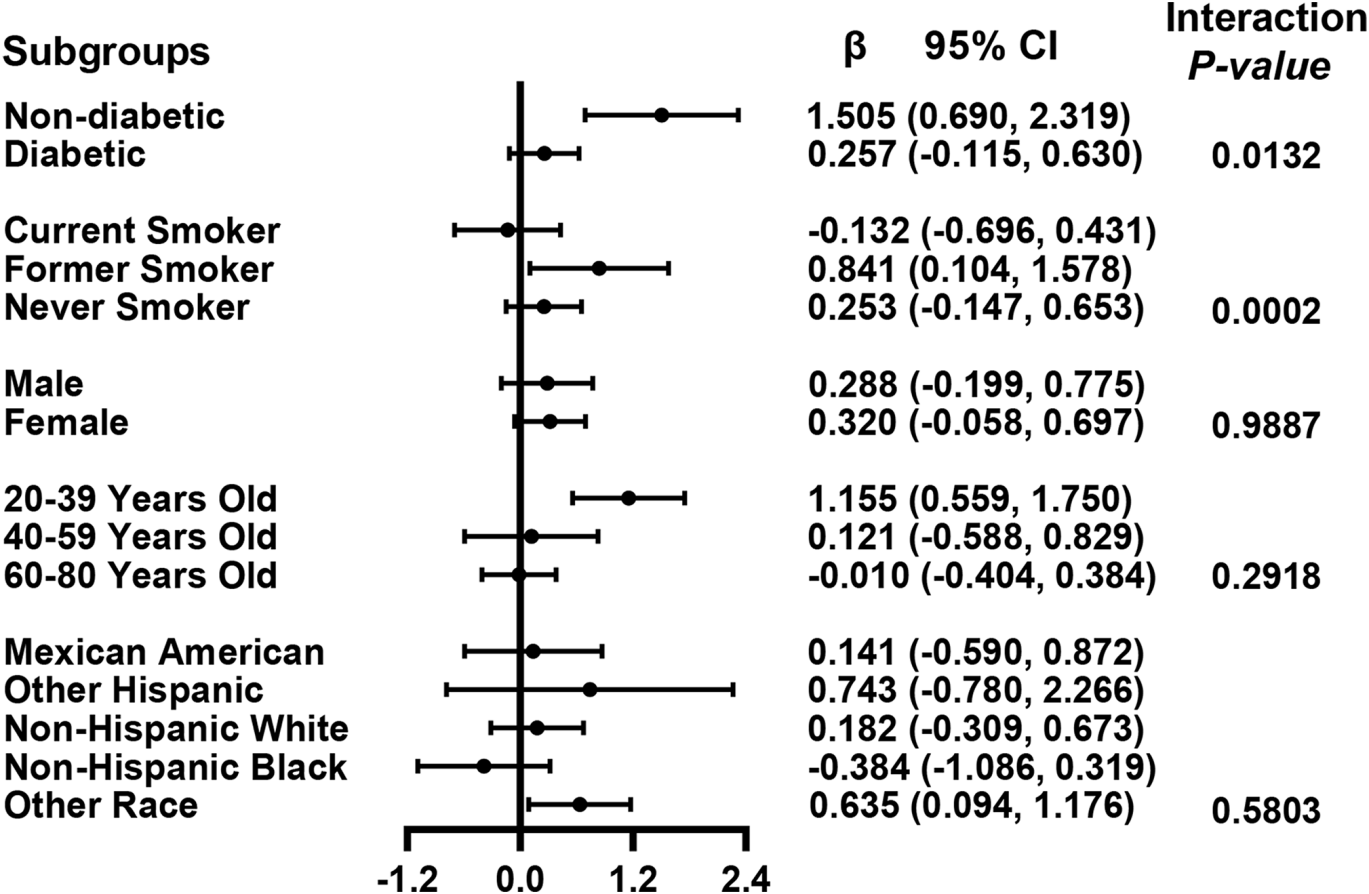
Association between glycohemoglobin and median LSM stratified by diabetes, smoking, age, gender and ethnicity. Each group was adjusted the following covariates except itself: Age, gender, ethnicity, body mass index, marital status, poverty, high education, physical activity, hypertension, smoking, alcohol use, alanine aminotransferase, aspartate aminotransferase, gamma glutamyl transferase, alkaline phosphatase, total cholesterol, total bilirubin, blood urea nitrogen and uric acid. LSM, Liver Stiffness Measurements; CI, Confidence Interval.

**Figure 4:**
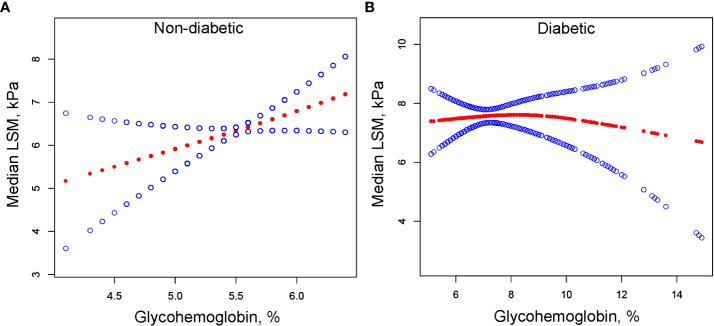
The correlation between glycohemoglobin and median LSM stratified by diabetes. **(A)** Non-diabetic subgroup. **(B)** Diabetic subgroup. The red line represents the smooth curve. The blue lines represent the 95% confidence interval. Age, gender, ethnicity, body mass index, marital status, poverty, high education, physical activity, hypertension, smoking, alcohol use, alanine aminotransferase, aspartate aminotransferase, gamma glutamyl transferase, alkaline phosphatase, total cholesterol, total bilirubin, blood urea nitrogen and uric acid were adjusted. LSM, Liver Stiffness Measurements.

**Figure 5:**
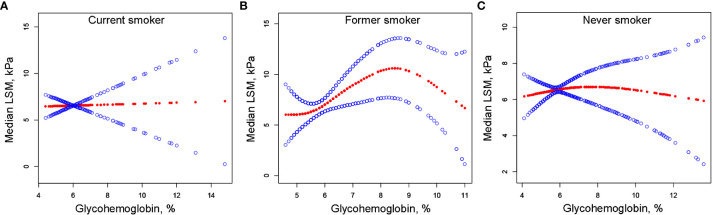
The correlation between glycohemoglobin and median LSM stratified by smoking. **(A)** Current smoker subgroup. **(B)** Former smoker subgroup. **(C)** Never smoker subgroup. The red line represents the smooth curve. The blue lines represent the 95% confidence interval. Age, gender, ethnicity, body mass index, marital status, poverty, high education, physical activity, hypertension, alcohol use, alanine aminotransferase, aspartate aminotransferase, gamma glutamyl transferase, alkaline phosphatase, total cholesterol, total bilirubin, blood urea nitrogen and uric acid were adjusted. LSM, Liver Stiffness Measurements.

We performed univariate analysis to find potential factors affecting the relationship between glycohemoglobin and median LSM (**Table S1**). Physical activity, smoking, AST, total cholesterol, and diabetes showed statistically significant correlation with median LSM. Then, the subgroup analysis of the above variables and age, gender, and ethics were conducted (**Figure 3**; **Table S2**). Among them, smoking and diabetes interact with glycohemoglobin. In the non-diabetic subgroup, glycohemoglobin had a positive correlation with median LSM (β=1.505, CI: 0.690-2.319). In the diabetic subgroup, the correlation between glycohemoglobin and median LSM was not statistically significant (β=0.257, CI: -0.115-0.630). **Figure 4** shows the smooth curve of the diabetes subgroup analysis. In the current smoker and never smoker subgroups, glycohemoglobin and median LSM had no statistically significant correlation (β=-0.132, CI: -0.696-0.431; β= 0.253, CI: -0.147-0.653). In the former smoker subgroup, there was a statistically significant positive correlation between glycohemoglobin and median LSM (β=0.841, CI: 0.104-1.578). The two-piecewise linear regression model also found a turning point at 8.6%. **Figure 5** shows the smooth curve of the smoking subgroup analysis.

## 4 Discussion

The present study analyzed the NHANES database of people ≥20 years old with NAFLD and found differences in median LSM values, hypertension rates, alcohol consumption, marriage rates and, physical activity rates between diabetic and non-diabetic individuals. We found a positive correlation between glycohemoglobin and median LSM using multivariable linear regressions and a saturating effect for this positive correlation. Then we used subgroup analysis to reveal an interaction between glycohemoglobin and diabetes and smoking status in the correlation between glycohemoglobin and median LSM.

Among patients with NAFLD, those with diabetes had higher median LSM values and hypertension prevalence than those without diabetes but were lower in daily alcohol consumption and had lower rates of marriage and physical activity. In a cross-sectional study of 557 patients with T2DM (mean age 61.4 ± 10.8 years), the investigators found a prevalence of NAFLD and advanced fibrosis of 72.4% and 21.0%, respectively, based on transient elastography (19). In another study of 561 patients with T2DM (mean age: 60 ± 11 years), the prevalence of steatosis was 70%, and the prevalence of fibrosis was 21% (20). The results of the present study also demonstrated that among patients with NAFLD, those with diabetes had higher liver stiffness values. It may be related to the fact that diabetes contributes to and accelerates the development of NAFLD (21). Cardiovascular disease and malignancy is the leading cause of death in NAFLD (15). In the present study, we found that the prevalence of hypertension was also increased in patients with NAFLD who had diabetes compared to those who did not have diabetes. Therefore, liver monitoring and blood pressure control should be taken seriously in NAFLD patients with diabetes. In addition, reducing alcohol consumption and increasing physical activity are also necessary.

In NAFLD patients, glycohemoglobin was positively correlated with median LSM, and this positive correlation disappeared when glycohemoglobin ≥8.6%. **Table 2** demonstrates that median LSM tended to increase with the increase of glycohemoglobin (*P*-trend<0.001), but they are not purely linearly correlated (*P*=0.07124). Then, we fitted their curves and found that the two actually showed a curvilinear correlation (**Figure 2**), and this curvilinear correlation was also found to have a saturation effect after using the two-piecewise linear regression model. Model 2 showed β=1.517 (P<0.00001) when glycohemoglobin<8.6% and β=-0.843 (P=0.12593) when glycohemoglobin≥8.6%. The trend test of Model 2 also showed that when glycohemoglobin <8.6%, median LSM showed an increasing trend with increasing glycohemoglobin (P-trend<0.001), and when glycohemoglobin≥8.6%, median LSM showed a decreasing trend with increasing glycohemoglobin, but this trend was not statistically significant (P-trend=0.072). Two large cohort studies found that 66% of patients over 50 years of age with diabetes or obesity had NASH with advanced fibrosis on index liver biopsy (22, 23). In addition, one study found that the carbohydrate-restricted diet (<20 g/d of carbohydrates) was more effective in reducing hepatic fat in patients with NAFLD compared to the reduced calorie diet (range, 1200-1500 kcal/d) (24). Therefore, we hypothesized that the glucose level is likely related to the progression of NAFLD. As one of the NAFLD stages, NASH is regulated by several mechanisms, including metabolic, genetic, environmental, and gut microbial factors (25). It has been suggested that alterations in glucose and lipid metabolism lead to hepatic steatosis and trigger an inflammatory environment that causes cellular damage in the liver and other tissues, followed by activation of hepatic stellate cells and collagen deposition, ultimately leading to fibrosis, cirrhosis, and even hepatocellular cancer (15). Changes in glycohemoglobin are indicative of the patient’s current glucose metabolic status. Therefore, the level of glycohemoglobin may also reflect the progression of NAFLD.

Pioglitazone is a thiazolidinedione antidiabetic drug, which can increase cellular sensitivity to insulin and thus lower blood glucose. Researchers have compared the efficacy of pioglitazone, vitamin E, and placebo in nondiabetic patients with NASH (PIVENS trial) and found that patients treated with pioglitazone had a higher proportion of complete resolution of steatohepatitis compared to those treated with placebo (47% vs. 21%) (26). Another study found that after 12 months of treatment with pioglitazone in nondiabetic subjects with NASH, subjects had improvements in liver injury and fibrosis (27). In addition, it has been demonstrated that thiazolidinediones can modestly improve fibrosis and hepatocellular ballooning, but it causes a significant increase in body weight (28). The results of the present study also suggest that those patients with lower glycohemoglobin had lower liver stiffness. Therefore, preventing high blood glucose levels may positively affect the treatment of NAFLD.

In the positive association of glycohemoglobin with median LSM, there was an interaction between glycohemoglobin and diabetes and smoking. We found no significant correlation between glycohemoglobin and median LSM in the diabetic subgroup, while they were positively correlated in the non-diabetic subgroup (**Figures 3**, **4**). The median LSM values were significantly higher in the diabetic subgroup than in the non-diabetic subgroup (7.757 ± 5.986 vs. 6.182 ± 5.076, P<0.00001). One of the reasons for this result was probably the different levels of glycohemoglobin in the two groups (non-diabetic: 5.526 ± 0.386 vs. diabetic: 7.535 ± 1.777, P<0.00001). As suggested by the results of the present study, the correlation between glycohemoglobin and median LSM was lost when the glycohemoglobin was ≥8.6%. In the former smoker subgroup, a positive correlation and threshold effect (at 8.6%) between glycohemoglobin and median LSM was observed, while no significant correlation was seen in the current smoker and never smoker subgroups. That may be related to each group’s different glycohemoglobin levels and distribution status. **Figure 5** shows that the distribution of glycohemoglobin in the former smoker subgroup was more dispersed, while the distribution of glycohemoglobin in the current smoker and never smoker subgroups was more skewed. In addition, a nine-year follow-up study demonstrated that the hazard ratios of diabetes in former smokers were 1.22 (CI, 0.99 to 1.50) compared to never smokers (29). Balkau and colleagues (30) analyzed DESIR (Data from an Epidemiological Study on the an Epidemiological Study on the Insulin Resistance Syndrome) cohort and found that a reduction of 10 cigarettes per day over 3 years was associated with s significant increases in insulin (7%), glucose (1.98 mg/dL), triglyceride level (8%), waist circumference (0.97 cm), and BMI (0.31 kg/m2) in men and with a significant increase in HDL cholesterol level (2.32 mg/dL), waist circumference (1.1 cm), and BMI (0.59 kg/m2) in women. Wannamethee and colleagues (31) found that smoking withdrawal resulted in significant weight gain in men, which may be related to increased appetite and excess caloric intake after withdrawal (32). Previous studies have also found obesity associated with inflammation in former smokers (33). Thus, the differences in smoking status may influence the relationship between glycohemoglobin and median LSM by affecting systemic metabolism, inflammation, and appetite.

## 5 Limitation

We must also acknowledge that the present study has some limitations. First, the present study is a cross-sectional study and cannot conclude a causal relationship between glycohemoglobin and median LSM. To prove their causal relationship and potential mechanisms, large sample cohort studies and basic experiments are necessary. Second, this study used the CAP score to diagnose NAFLD, which is not the gold standard and is susceptible to interference by severe obesity, acute hepatitis, hepatic iron overload, or the presence of ascites (7). However, liver biopsy is unsuitable for mass screening due to its invasive nature. Third, although we have adjusted for several variables in the model 2, there may still be other relational variables affecting median LSM and glycohemoglobin that we have not considered. Fourth, in terms of variable definition, there may be potential bias due to the subjective nature of the questionnaires used.

## 6 Conclusion

Overall, the present study found a positive association between glycohemoglobin and median LSM in patients aged 20-80 with NAFLD, and this positive association disappeared after glycohemoglobin ≥8.6%. Diabetes and smoking status can affect the relationship between glycohemoglobin and median LSM. Therefore, we hypothesized that glycohemoglobin may be a potential marker of NAFLD progression, and prevention of excessive glycohemoglobin may be beneficial in delaying NAFLD progression, especially in non-diabetic and former smokers. In addition, we recommend that patients with NAFLD with diabetes pay more attention to their liver, blood pressure and marital status and increase physical activity appropriately. However, more high-quality cohort studies with large samples are still needed to confirm this research issue further.

## Data availability statement

Publicly available datasets were analyzed in this study. This data can be found here: https://wwwn.cdc.gov/nchs/nhanes/Default.aspx.

## Ethics statement

Ethical review and approval was not required for the study on human participants in accordance with the local legislation and institutional requirements. The patients/participants provided their written informed consent to participate in this study.

## Author contributions

JH was responsible for data collection and processing and part of the writing; YL and ZD were responsible for part of the writing of the paper; JS and MZ were responsible for the design of the study. All authors contributed to the article and approved the submitted version.
