# Supplementary material for: Glycohemoglobin: A new warning strategy for non-alcoholic fatty liver disease: Study from the NHANES 2017- 2020
Source: Front Endocrinol (Lausanne). 2022 Dec 22;13:1078652. doi: 10.3389/fendo.2022.1078652 (PMC9813781; doi:10.3389/fendo.2022.1078652)
Supplement: Supplementary file 1 [file Table_1.docx]

Supplementary Material

**Table S 1 The results of univariate analysis.**

|  | **Statistics (N=1510)** | | **β (95% CI)** | ***P* Value** |
| --- | --- | --- | --- | --- |
| Age, y | 53.672 ± 18.205 | | -0.002 (-0.017, 0.013) | 0.79357 |
| Ethnicity, % |  |  | |  |
| Mexican American | 164 (10.861%) | 0 (Reference) | |  |
| Other Hispanic | 165 (10.927%) | 0.208 (-1.125, 1.541) | | 0.75974 |
| Non-Hispanic White | 504 (33.377%) | 0.208 (-0.825, 1.241) | | 0.69339 |
| Non-Hispanic Black | 454 (30.066%) | 0.462 (-0.747, 1.671) | | 0.45381 |
| Other Race | 223 (14.768%) | -0.047 (-1.366, 1.272) | | 0.94416 |
| Gender |  |  | |  |
| Male | 722 (47.815%) | 0 (Reference) | |  |
| Female | 788 (52.185%) | 0.010 (-0.559, 0.579) | | 0.97334 |
| Body mass index | 30.305 ± 7.921 | -0.004 (-0.041, 0.034) | | 0.85439 |
| Marital Status |  |  | |  |
| Unmarried | 737 (48.808%) | 0 (Reference) | |  |
| Married | 773 (51.192%) | -0.079 (-0.649, 0.490) | | 0.78470 |
| Poverty status |  |  | |  |
| Non-poverty | 1031 (68.278%) | 0 (Reference) | |  |
| Poverty | 479 (31.722%) | -0.404 (-1.051, 0.244) | | 0.22193 |
| Education status |  |  | |  |
| Non-high education | 382 (25.298%) | 0 (Reference) | |  |
| High education | 1128 (74.702%) | -0.122 (-0.881, 0.636) | | 0.75205 |
| Physical activity |  |  | |  |
| Inactive | 412 (27.285%) | 0 (Reference) | |  |
| Active | 1098 (72.715%) | -0.922 (-1.536, -0.308) | | 0.00329 |
| Hypertension |  |  | |  |
| Non-hypertension | 756 (50.066%) | 0 (Reference) | |  |
| Hypertension | 754 (49.934%) | -0.003 (-0.580, 0.574) | | 0.99113 |
| Smoking |  |  | |  |
| Current smoker | 266 (17.616%) | 0 (Reference) | |  |
| Former smoker | 382 (25.298%) | 1.722 (0.874, 2.570) | | 0.00007 |
| Never smoker | 862 (57.086%) | 0.330 (-0.421, 1.082) | | 0.38937 |
| Alcohol intake, g/d | 9.635 ± 29.155 | -0.009 (-0.020, 0.001) | | 0.07108 |
| ALT, u/l | 20.577 ± 15.251 | 0.005 (-0.015, 0.025) | | 0.60661 |
| AST, u/l | 21.290 ± 12.320 | 0.028 (0.002, 0.054) | | 0.03516 |
| GGT, iu/l | 30.060 ± 34.250 | 0.009 (-0.001, 0.018) | | 0.08985 |
| ALP, iu/l | 80.999 ± 26.794 | -0.006 (-0.017, 0.006) | | 0.33342 |
| Total bilirubin, umol/l | 7.478 ± 4.642 | -0.022 (-0.081, 0.036) | | 0.45663 |
| Total cholesterol, mmol/l | 4.811 ± 1.069 | -0.282 (-0.555, -0.009) | | 0.04307 |
| Blood Urea Nitrogen, mmol/l | 5.512 ± 2.259 | 0.049 (-0.092, 0.190) | | 0.49516 |
| Uric Acid, umol/l | 328.315 ± 92.316 | -0.000 (-0.003, 0.003) | | 0.87837 |
| Diabetes |  |  | |  |
| Non-diabetic | 1113 (73.709%) | 0 (Reference) | |  |
| Diabetic | 397 (26.291%) | 1.575 (0.928, 2.222) | | <0.00001 |

Mean±SD for continuous variables; % for Categorical variables. ALT, Alanine Aminotransferase; AST, Aspartate Aminotransferase; GGT, Gamma Glutamyl Transferase; ALP, Alkaline Phosphatase; CI: Confidence Interval.

**TableS 2 Association between glycohemoglobin and median LSM stratiﬁed by total cholesterol, AST and physical activity**

|  | **Crude Model** | **Model 1** | **Model 2** |
| --- | --- | --- | --- |
|  | **β (95% CI)**  ***P* Value** | **β (95% CI)**  ***P* Value** | **β (95% CI)**  ***P* Value** |
| Total cholesterol, mmol/l (Quartile) |  | | |
| Q1 | 0.983 (0.444, 1.523)  0.00041 | 0.869 (0.320, 1.419)  0.00210 | 0.845 (0.078, 1.612)  0.03167 |
| Q2 | 0.554 (0.207, 0.900)  0.00189 | 0.556 (0.207, 0.906)  0.00196 | 0.231 (-0.246, 0.708)  0.34279 |
| Q3 | 0.235 (-0.063, 0.532)  0.12336 | 0.243 (-0.059, 0.546)  0.11548 | 0.213 (-0.209, 0.635)  0.32339 |
| Q4 | 0.336 (-0.210, 0.883)  0.22843 | 0.325 (-0.227, 0.877)  0.24877 | -0.220 (-1.040, 0.601)  0.60002 |
| *P*-interaction | 0.5050 | 0.4884 | 0.5408 |
| AST, u/l (Quartile) |  | | |
| Q1 | 0.324 (-0.286, 0.935)  0.29835 | 0.311 (-0.304, 0.927)  0.32266 | 0.250 (-0.622, 1.121)  0.57505 |
| Q2 | 0.184 (-0.133, 0.501)  0.25530 | 0.162 (-0.158, 0.482)  0.32108 | 0.071 (-0.391, 0.534)  0.76197 |
| Q3 | 0.944 (0.486, 1.402)  0.00007 | 0.911 (0.448, 1.374)  0.00014 | 0.737 (0.056, 1.417)  0.03457 |
| Q4 | 0.547 (0.114, 0.980)  0.01367 | 0.527 (0.090, 0.964)  0.01872 | 0.031 (-0.650, 0.712)  0.92964 |
| *P*-interaction | 0.3404 | 0.3252 | 0.1732 |
| Physical activity |  | | |
| Inactive | 0.477 (0.032, 0.922)  0.03620 | 0.482 (0.031, 0.934)  0.03706 | 0.237 (-0.368, 0.841)  0.44389 |
| Active | 0.473 (0.228, 0.718)  0.00017 | 0.466 (0.220, 0.713)  0.00022 | 0.380 (0.032, 0.728)  0.03275 |
| *P*-interaction | 0.9922 | 0.9781 | 0.8226 |

Crude Model: No covariates were adjusted. Model 1: Age, gender and ethnicity were adjusted. Model 2: Age, gender, ethnicity, body mass index, marital status, poverty, high education, physical activity, hypertension, smoking, alcohol use, alanine aminotransferase, aspartate aminotransferase, gamma glutamyl transferase, alkaline phosphatase, total cholesterol, total bilirubin, blood urea nitrogen and uric acid were adjusted (except for the analyzed variable itself). LSM, Liver Stiffness Measurements; AST, Aspartate Aminotransferase; CI: Confidence Interval.
